# Applying of Network Robustness Index (NRI) for prioritization inner-city of Tabriz metropolis ‎in disaster situation 

**Published:** 2019-08

**Authors:** Ahad Moghaddamsafaeiazar

**Affiliations:** ^*a*^Crisis Management Organization of Metropolitan Municipality of Tabriz.

## Abstract

Natural and non-natural disasters have harmful results in various field of traffic and ‎transportation configuration. Infrastructure performance have a huge impact in disaster situation ‎and can increase or decrease effects of damage. Considering the unpredictable feature intensity ‎of disaster, appropriate decisions can reduce failure. In this paper, emphasis of sample ‎surveying for operating traffic and transportation network with regard to, network link capacity ‎for access to various disaster situation. For that, case of TABRIZ inner-city highway network, ‎Investigation the important links based on Network Robustness Index (NRI). So Tabriz inner-‎city networks links, are disconnect in each iteration and OD matrix assigned. Aggregate results ‎on network such as Vehicle Hour Trip VHT are calculated and used for prioritization. Based on ‎reduced VHT, links are categorized in 4 classes. Very much disaster, Very disaster, Moderate ‎disaster and low disaster.‎

**Keywords:**

Prioritization, Disaster Management, Assignment, Capacity, Volume, Highway, VHT, NRI.‎

